# *R2Play* development: Fostering user-driven technology that supports return-to-play decision-making following pediatric concussion

**DOI:** 10.3389/fresc.2022.1051579

**Published:** 2022-12-05

**Authors:** Danielle DuPlessis, Emily Lam, Fanny Hotze, Ajmal Khan, Hiba Al-Hakeem, Stephanie McFarland, Andrea Hickling, Michael Hutchison, F. Virginia Wright, Nick Reed, Elaine Biddiss, Shannon E. Scratch

**Affiliations:** ^1^ Bloorview Research Institute, Holland Bloorview Kids Rehabilitation Hospital, Toronto, ON, Canada; ^2^ Rehabilitation Sciences Institute, Temerty Faculty of Medicine, University of Toronto, Toronto, ON, Canada; ^3^ Temerty Faculty of Medicine, Institute of Biomedical Engineering, University of Toronto, Toronto, ON, Canada; ^4^ Department of Occupational Science and Occupational Therapy, Temerty Faculty of Medicine, University of Toronto, Toronto, ON, Canada; ^5^ Faculty of Kinesiology and Physical Education, University of Toronto, Toronto, ON, Canada; ^6^ Department of Physical Therapy, Temerty Faculty of Medicine, University of Toronto, Toronto, ON, Canada; ^7^ Department of Paediatrics, Temerty Faculty of Medicine, University of Toronto, Toronto, ON, Canada

**Keywords:** pediatrics, concussion, assessment, user-centered design, sport medicine, return-to-play

## Abstract

**Objective:**

To design a multi-domain return-to-play assessment system (*R2Play*) for youth athletes with concussion.

**Methods:**

The *R2Play* system was developed using an overarching user-centered approach, the Design Thinking Framework, and research activities included: 1) structured brainstorming within our research team, 2) interviews with clinician and youth sports coaches, 3) building a testable prototype, and 4) interface testing through cognitive walkthroughs with clinician partners.

**Results:**

Clinician and coach participants provided feedback on the *R2Play* concept, which was integrated into the design process and provided future directions for research. Examples of feedback-driven design choices included reducing assessment time, increasing ecological validity by adding in background noise, and developing youth-friendly graphical results screens. Following refinement based on stakeholder feedback, the *R2Play* system was outlined in detail and a testable prototype was developed. It is made up of two parts: a clinician tablet, and a series of tablet “buttons” that display numbers and letters. Youth athletes run between the buttons to connect a “trail” in ascending alphanumeric order, 1-A-2-B, etc. Their performance across a series of levels of increasing difficulty is logged on the clinician tablet. Initial testing with five clinicians showed the system's interface to have excellent usability with a score of 81% (SD = 8.02) on the System Usability Scale.

**Conclusion:**

Through this research, a prototype of the *R2Play* system was innovated and evaluated by clinician and coach stakeholders. Initial usability was excellent and directions for future iterations were highlighted. Outcomes suggest the potential benefits of using technologies to assist in complex clinical assessment, as well as utilizing a user-centered approach to design.

## 1. Introduction

Pediatric concussion is a serious public health concern. While estimating the burden of these injuries is a challenge due to variable access to care and underreporting, a recent study on a Canadian sample estimates that more than 2000 per 100,000 youth will experience concussion annually (1, 2). Sport is a major cause of concussion among youth people aged 11–19 years; per United States data, approximately 41% of all concussions in this age group are sustained while playing sports (3).

Compared to adults, youth athletes may also be more susceptible to repeat concussion and are more likely to experience prolonged recovery (4). However, this population is historically underrepresented in research, resulting in few age-appropriate measures of assessment for youth (4). Many practices in pediatric concussion are carried over from adult populations, despite the fact that children and adolescents are not “little adults”: from the pathophysiology of the developing brain, to their neurobehavioral outcomes, to their return-to-activity goals, youth are unique (5, 6).

The consequences of misdiagnosis or inadequate treatment of concussion in youth athletes can be severe, ranging from prolonged recovery, to repeat concussions, to subsequent musculoskeletal injury, and, in extremely rare cases, death (6–11). As such, determining when the brain has recovered from concussion and athletes are ready to return to the playing field is of critical importance. While no youth-specific guidelines exist, the current concussion consensus statement best practice guidelines for youth and adult return-to-play recommend a multi-modal approach to assessment, which incorporates symptom self-reporting, neuropsychological, and physiological measures (12). This is typically carried out as a battery of single-domain assessments, where processes affected by concussion (e.g., balance, memory, exertion) are assessed in isolation (13).

Sport, however, is not a single-domain activity. Rather, it involves a complex multi-domain environment that requires integration of physical, cognitive, and socio-emotional skills (14–17). While performance on single-domain assessments may return to pre-injury levels as early as 7–10 days post-injury, a growing body of literature suggests that subclinical deficits persist and can be elicited by more complex tasks. These subtle differences in gait, postural control, and cognitive processing may have serious consequences if they manifest on the playing field (8, 16, 18–22).

Emerging research calls for implementation of more ecologically relevant, multi-domain assessments of concussion prior to return-to-play (23). While there has been an increase in the use of multi-domain assessments in research, barriers exist to translating this work into clinical practice (24). Current assessment paradigms often fall short of effectively simulating sport, and are challenging to implement and score (16, 24–27).

We aimed to bridge this gap by working collaboratively with stakeholders to develop a user-driven multi-domain assessment (*R2Play*) to help bolster return-to-play decision making following pediatric concussion. Ultimately, following rigorous testing, we hope that this multi-domain assessment will help promote the safety of youth athletes, and allow clinicians, athletes, and parents to have confidence in return-to-play clearance. This paper describes the co-creation process undertaken to develop the *R2Play* system along with usability testing of its clinician interface. To guide this work, we adopted the *Design-Thinking Framework* (28). This framework is a lens through which a problem can be understood and solved by working through a series of iterative modes: empathize, define, ideate, prototype, and test (28, 29). A design-thinking mindset places empathy with end-users and meaningful collaboration at the forefront (30) and is considered especially applicable to the kinds of complex, multidimensional challenges often seen in a healthcare context (30). This approach can encompass diverse research activities, applying a multi-method approach to fostering research innovation (30). Examples include:“needfinding” (i.e., defining the problem and identifying user priorities) through reviewing current approaches, conducting environmental scans, and interviewing end-users; brainstorming both within the research group and by seeking out opinions from multidisciplinary partners; and rapid prototyping in collaboration with end-users (31). Aligned with this framework, our three research objectives were:

• **Objective One**: to conduct (a) a scoping review (24), (b) discussion and brainstorming within our interdisciplinary design team composed of key partners and (c) qualitative interviews to capture broader stakeholder feedback for the purposes of *empathizing* with end users, *defining* our problem, and *ideating* on possible solutions.
• **Objective Two**: to build a *prototype* of the *R2Play* system. This involved collaboratively refining our ideas and incorporating stakeholder feedback to move from low, to medium, then high-fidelity prototypes.
• **Objective Three**: to *test* the *R2Play* system prototype. Of note, validation of *R2Play* is anticipated to be a multiyear iterative process. In this paper, we describe usability testing of the *R2Play* software interface conducted *via* cognitive walkthroughs with clinician stakeholders.

This paper is organized with respect to the three research objectives with separate methods and results section for each, followed by an integrated discussion section. Ethics approvals for all studies were obtained from the Holland Bloorview Kids Rehabilitation Hospital (HBKRH) Research Ethics Board (REB #19–855). Due to restrictions associated with the COVID-19 pandemic, all phases of this research were carried out virtually using a videoconferencing platform.

## 2. Objective one: Empathize, define, and ideate

### 2.1 Problem definition and early ideation

This project emerged from a clinician-identified desire for more comprehensive assessment tools to increase confidence in return-to-play decision making for youth athletes post-concussion. An interdisciplinary team of health practitioners, engineers, athletes, and researchers was established to address this need. Knowledge-sharing activities were carried out amongst the team. Clinical partners demonstrated how return-to-play assessments are currently carried out at HBKRH (e.g., neurocognitive tests, balance and endurance tests), engineering partners showcased different technologies that could be used to support assessments (e.g., 3D depth sensors, tablets, inertial sensors), and youth and family leaders with a history of concussion described their experiences. A scoping review, reported in detail in another paper (24), of sixty-four articles describing 36 unique assessments was conducted. Overall, this scoping review revealed (i) a lack of assessments that effectively simulated the speed and complexity at which cognitive processing occurs in sport, and, (ii) multiple barriers to clinical implementation of multidomain assessments including cost, set-up time, space, and the cognitive load on clinicians while scoring (24). Guided by learnings from the scoping review, knowledge sharing activities, and the clinical experience of our interdisciplinary team, a set of design objectives for a novel return-to-play assessment were established (Table 1).

**Table 1:** Design objectives for R2Play system.

Objective	Description	Rationale
Sport-like	Task (s) should require integration across physical, cognitive, and socio-emotional domains while responding to a dynamic environment.	Aligns with the core components of sport previously identified in the literature (14–16). Also addresses gaps in current multi-domain assessment paradigms (24).
Fun for youth athletes	Task (s) should be engaging and fun for youth athletes 10–25 years old.	Fostering engagement in rehabilitation improves motivation and contributes to more positive outcomes and experiences for children and adolescents (32).
Easy to use	Assessment should be easy for youth athletes to understand.	Technology that is simple and “user-friendly” is more likely to be adopted into clinical practice (33).
	System should minimize the cognitive load on clinicians during both administration and scoring.	High cognitive load for clinicians due to the complexity of administering and scoring current multi-domain assessments has created a barrier to translating this research into practice (24, 27).
Low-cost	System should be at an accessible price point for clinicians. The technology must be as low-cost as possible while maintaining functionality.	High-cost technology presents a barrier to implementation for clinicians; an expensive tool may not be adopted as willingly or as widely (33).
Flexible	System should be able to be used in different clinical spaces, and customizable to suit the individual ages and abilities of youth athletes.	Incompatibility with existing practice is a known barrier for integration of technology into clinical practice (33).
Clinically informative	Should provide clinicians with useful (valid and reliable) data to inform their return-to-play decision making.	Evidence of high utility is a facilitator for implementation of technology into clinical practice (33).

**Figure 1:**
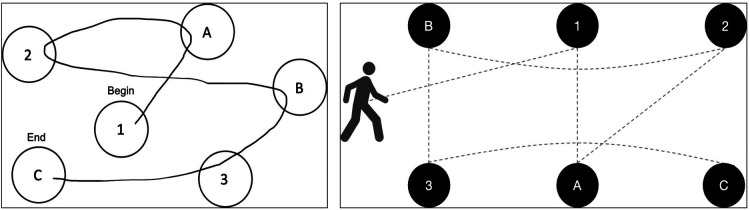
The Trail Making Test (left), and a diagram depicting the embodied *R2Play* task concept (right).

These design objectives guided early brainstorming and ideation from which two themes emerged: 1) the potential use of technology to support clinicians in administering and scoring more complex sport-like assessments; and, 2) the idea of integrating components of existing neuropsychological tests that clinicians are familiar with (e.g., Color-Word Interference (34), Trail Making (34), Contingency Naming (35), Digit Span (34)) into a sport-like activity. Further discussion with clinicians and amongst the team identified the Trail Making Test as a promising candidate to serve as the cognitive backbone of *R2Play*, due to its loading on fluid cognitive abilities, working memory, and processing speed (36). Traditionally, the Trail Making Test is completed by connecting numbers and/or letters in sequence using a pencil on paper (37). The task is easy to explain and routinely used by clinicians to assess executive functioning and neuropsychological impairment in children and adults (38, 39). In our early conceptualization, we envisioned recreating the test in a physical space wherein athletes connect the trail by running and pressing buttons displaying the letters and numbers. With this concept in mind, we created a series of rough prototype drawings to present to stakeholders for feedback and refinement (Figure 1).

### 2.2 Qualitative interviews to refine problem definition and ideation

To engage with stakeholders more broadly, we conducted a series of semi-structured qualitative interviews. Two groups were included in the interviews: clinicians, who are the target end-users of the R2Play system, and youth sports coaches, who are experienced in designing ecological sports drills and engaging with youth athletes. The research question guiding this inquiry was: “How can we design the *R2Play* system to best meet the needs of clinician end-users?”. Notably, we anticipate that the *R2Play* system is most relevant for youth athletes returning to open-skill sports which are among the highest risk for concussion and require a high degree of multi-domain processing (40, 41). As such, coaches were primarily recruited from open-skill sport backgrounds.

#### 2.2.1 Sampling and recruitment

We sampled purposively to seek out information-rich stakeholders and worked deliberately to include diverse perspectives (42). As interviews were carried out virtually, clinicians and youth sports coaches were recruited from across Canada. Inclusion criteria: clinicians – ≥ one year of experience working with children and adolescents (6–19 years) who had sustained a concussion; coaches – ≥ one year of experience coaching youth (6–19 years) in organized sport. Participants were recruited until the sample had sufficient information power to address our research question, which corresponded to 6 clinicians and 4 youth sports coaches (43).

#### 2.2.2 Data collection

Semi-structured interviews (carried out by DD) were conducted over the online videoconferencing platform Zoom (44). Audio was recorded for review. Interviews began by exploring experiences with return-to-play broadly. Next, the interviewer shared their screen with participants and presented a brief demonstration of the low-fidelity prototype using Microsoft PowerPoint (45). Afterwards, participants were encouraged to share their impressions and give targeted feedback on the assessment concept. See Supplementary Materials Table S2 for complete interview guide. Following each interview, a reflexive analytic audio memo was recorded by the interviewer (DD) describing impressions and insights.

#### 2.2.3 Data analysis

Interview data were analyzed using content analysis (46). Codes were derived inductively from the data without imposing a theoretical framework (46). In doing so, we helped ensure that the codes were grounded in the participant's experiences (46). The first three interview transcripts were read by two reviewers (DD, EL) to familiarize themselves with the data. During this time, the interviews were pre-coded by highlighting key ideas, important passages, and potential codes (47). Analytic memos also provided context to this pre-coding process. After transcripts were reviewed, the reviewers fully coded the first three interviews independently. Whenever possible, concepts were coded “in vivo”, using participants' own words as codes (47). Next, codes were discussed in team meetings, and then codes, their definitions, and instances of the codes were gathered in an iterative codebook. The codebook formed the beginning of an audit trail, which tracked the evolution of data from codes, to categories, to themes, to study findings (47, 48). The codes from the first three interviews were then transitioned into NVivo qualitative data analysis software (49), which facilitated the coding of the remainder of the dataset (49). Coding was a cyclical process, such that as new codes were added, earlier interviews were iteratively re-coded. Strategies for rigour consisted of collaborative coding of the first three interviews (DD, EL), verification of codes by a team member (EL), documentation of analysis using an iterative code book, and discussions of codes and themes within the research group. This analysis led to the creation of a “design table” summarizing stakeholder-identified considerations for the *R2Play* system and prototype. For each consideration, potential design recommendations and revisions were brainstormed and included to track the iterative co-creation process. Proposed design revisions were categorized as: implemented (i.e., incorporated into the *R2Play* system prototype), iterating (i.e., compatible with the *R2Play* concept, but not yet integrated), or incompatible. While not all feedback could be implemented in the first iteration of the *R2Play* design, the “design table” provided a medium to explain which pieces were addressed, why, and how. Additionally, we documented the feedback that we were unable to incorporate to increase the transparency of our work.

#### 2.2.4 Results

Table 2 summarizes demographics of the 10 participants.

**Table 2:** Stakeholder participant characteristics.

Age Range (Years)	Gender	Discipline	Sector	Years of Experience
25–34	F	Occupational Therapist	Public and Private	5
35–44	F	Athletic Therapist	Public	14
25–34	F	Specialized Neurorehabilitation Athletic Therapist and Physiotherapist	Private	10
45–54	F	Physiotherapist	Private	26
45–54	F	Trauma Coordinator and Physiotherapist	Public	31
35–44	M	Chiropractor	Private	10
Age Range (Years)	Gender	Discipline	Age of Athletes	Years of Experience
55+	M	Coach (*Basketball*, Football)	15–19	40
25–34	F	Coach (*Ice Hockey*, Basketball, Soccer)	13–19	14
35–44	M	Coach *(Soccer)*	13–18	20
35–44	M	Coach (*Rugby*, Basketball)	4–45	16

For coach participants, their primary sport is italicized where applicable.

Feedback collected during the interviews was positioned within the following constructed themes: 1) the individuals being assessed, 2) the *R2Play* system (assessment tasks, system hardware, clinician software interface, scoring), and 3) clinic integration (time, space, training). Detailed feedback and resultant design iterations are presented in the “design table” (See Supplementary Materials Table One). Here we summarize stakeholder feedback with respect to each of the above themes broadly, and focus on feedback that was integrated into the early *R2Play* prototype.

##### 2.2.4.1 Individuals being assessed

In thinking about the *individuals being assessed*, stakeholders indicated the importance of designing for accessibility (e.g., for wheelchair users, individuals with colour blindness, dyslexia or other learning disabilities, language) and for diverse ages. Understanding each athletes' individual context was also a stakeholder priority (e.g., concussion history, psychosocial influences, school, symptoms before/after, mental health, sport training profile, co-morbidities). Comments relating to capturing individual contexts were incorporated into the *R2Play* system through an optional pre-assessment client profile, where clinicians are guided through a detailed clinical history. To improve accessibility, the *R2Play* tablets were placed in raised stands. Pending promising usability and psychometric data, future work will strive to further improve accessibility of the *R2Play* system and to adapt our system to be suitable for younger children.

##### 2.2.4.2 The *R2Play* system

With respect to the *R2Play* system, feedback was provided on early ideation of the *R2Play* tasks, hardware, software interface, and scoring:

• Tasks: The *R2Play* tasks were considered sport-like by coach stakeholders who served as our “ecological validity experts,” taxing physical skills like speed, agility, and hand-eye coordination; perceptual skills like visual scanning and auditory cues; cognitive skills like planning and decision-making; and socio-emotional aspects associated with scoring/competition in sport. Stakeholders also introduced the need for dynamic decision-making and reaction (perception-action integration) and the possibility of auditory interference in line with a noisy sporting environment.
• Hardware: User-friendliness and durability were important components of system hardware.
• Software interface: Key stakeholder-identified considerations for the system software interface were the need for child-friendly performance reports as well as an interface for clinicians to document observational notes.
• Scoring: Lastly, scoring and interpretation of the *R2Play* assessment was a prominent theme. There was interest in establishing clinical normative data and also in establishing target heart rate zones for exertion. There was also interest in automatically logging errors and reaction times to support clinical interpretation and decision-making.

The addition of background noise and a scramble condition stresses perception-action integration were implemented (see Section 3.1 for detail). Soft rubber cases were added to increase the durability of the *R2Play* tablets. To assist in recording observations and errors, a virtual notepad was developed to be used by clinicians on the *R2Play* interface. Results screens were designed to be graphic and simple, to assist in communicating assessment findings with youth clients.

Not all user feedback was able to be implemented. At this time, the task does not include sport-specific implements (e.g., sticks, balls) or contact/physicality (these may be incorporated in future iterations). Additionally, it does not simulate communication with teammates or coaches, which may also be an area for future development. Next steps for the *R2Play* system will also be focused on contextualizing and interpreting *R2Play* results, and understanding the psychometric properties of the assessment.

##### 2.2.4.3 Clinic integration

Lastly, in providing feedback on *R2Play* and considering needs for a multidomain return-to-play assessment, the theme of *clinic integration* emerged from the data and in particular, issues of timing, space, and training. Clinician feedback indicated a preferred assessment time of <30 min. The ability to set-up *R2Play* in different spatial configurations (e.g., a gym, a long hallway) was also expressed. Understanding the optimal spatial arrangement that can accommodate space constraints while still providing a sport-like, physically taxing experience was considered an important area of research and development by our stakeholders. Lastly, the importance of providing training resources both to navigate system setup and use, as well as for scoring/interpretation was emphasized. Comments relating to clinicians' use of time were especially salient, and *R2Play* levels were streamlined to shorten the duration of the assessment. A flexible system was developed to accommodate different assessment spaces, although additional research is required to understand impacts of different layouts on scoring/interpreting *R2Play* performance.

## 3. Objective two: Prototype design and build

**Figure 2:**
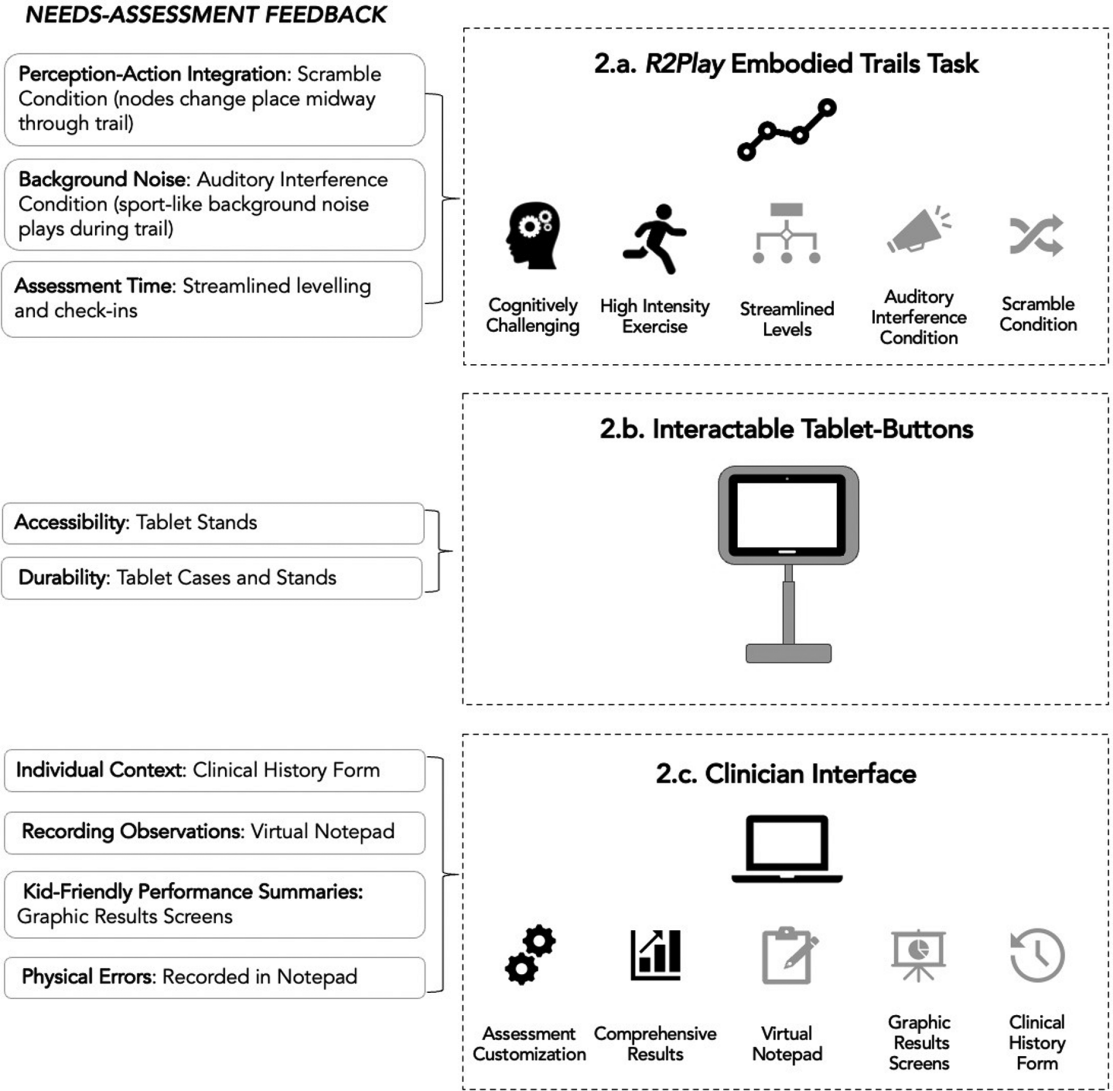
Integration of interview feedback into a second iteration of the *R2Play* prototype. Black icons indicate priorities that were identified and integrated into the *R2Play* concept prior to interviews, while grey icons indicate changes made following stakeholder feedback.

Following analysis of objective one interviews, our team (DD, EL, AK, FH) set out to build a testable high-fidelity *R2Play* prototype that incorporated user feedback. Figure 2 depicts some of the design iterations made and how they relate to the themes described above. The following objective two sections describe the design process and updated prototype with respect to key elements of the *R2Play* system, namely: (i) the task itself, (ii) the clinician software interface for setting up and administering the task, (iii) the hardware interface, and (iv) the scoring system (12).

### 3.1 Task refinement

**Figure 3:**
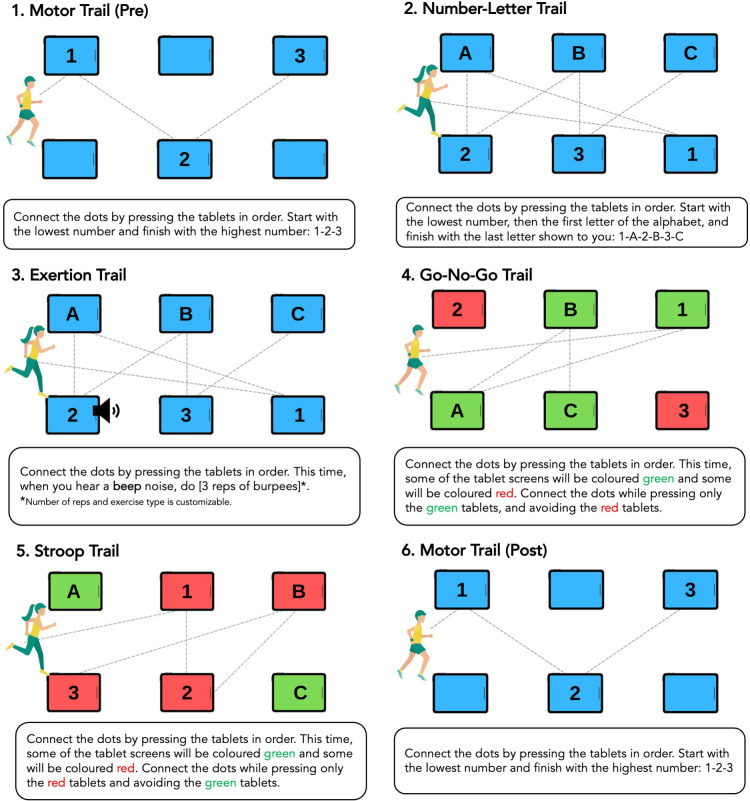
*R2Play* levelling.

Guided by objective one findings, the *R2Play* assessment was designed to include levels of varying physical effort and cognitive complexity, plus a pre- and post- motor task. The length of the assessment is customizable to either 6, 12, or 18 nodes in the trail with four levels: number-letter trail, exertion trail, go/no-go trail, and Stroop trail, depicted in Figure 3. The most basic level, the *number-letter trail*, is accomplished by pressing tablets in order (1-A-2-B-3-C). During the *exertion trail*, a sound cues participants to stop and complete a set of exercises (e.g., 3 burpees) before completing the trail. The type of exercise completed and the number of repetitions is also customizable, to suit the age and ability of the athlete. The *go-no-go trail* requires participants to exercise inhibitory control by completing the trail using only green numbers/letters and excluding any that are red. This rule is then reversed in the *Stroop trail*, where participants must select only the red numbers/letters and exclude any that are green. Each athlete completes four repetitions per level. Two of these repetitions have layered challenges: a scramble condition where the letters/numbers change places midway and the participant must adapt in a way that taxes perception-action integration, and an auditory interference condition where background noise play (e.g., gym sounds, cheering). A video demonstration is available. Each level begins with a training session carried out by the clinician interface software, followed by multiple repetitions, and ends with a brief check-in of perceived exertion and concussion symptoms. Additionally, it was recognized that a symbol trail could be preferable for younger children or children with different learning needs (e.g., dyslexia) and this will be considered for future extensions.

### 3.2 Clinician software interface

To assist in translating *R2Play* from a concept to a working prototype, a hierarchical task analysis (50) was conducted to build out an in-depth breakdown of the *R2Play* assessment structure and workflow. Following the established roadmap, team members (DD, EL) designed the graphical user interface for *R2Play*'s clinician interface. This preliminary prototype consisted of a series of low-fidelity wireframes, which were two-dimensional illustrations of the layout of content clinicians would see while administering the task. The goal for the interface was to create a simple, user-friendly means of working through the *R2Play* assessment. Key functionalities for the interface include: connecting to the tablet-buttons, creating an athlete profile with optional fields for documenting a detailed history, creating an assessment layout, collecting a pre-assessment resting heart rate and adolescent postconcussion symptom inventory (51), training youth athletes on rules of the *R2Play* task, carrying out the assessment, and recording results, including functionality to document clinical observations and display results *via* child-friendly visuals. The graphical user interface and described functionalities were implemented by one of our engineers (FH) using Unity and C#.

### 3.3 Hardware interface

The stakeholder team engaged in collaborative brainstorming exercises to develop a list of interactive technologies that could be used to support this novel return-to-play assessment concept with reference to our system design objectives (see Table 1). Using low-cost tablets as “buttons” was determined to be a suitable option due to their cost-effectiveness and flexibility to serve as a display to guide the task, as well as an interface which can register, log, and communicate participants' inputs throughout the task. We decided to implement a six-tablet system which would support varying trail lengths. Of note, for trails longer than 6 nodes, the tablet displays refresh after the first 6 nodes are completed so that the athlete can double-back to continue the trail. The final R2Play hardware configuration consisted of six low-cost Android tablet (e.g., Lenovo Tab E10) with cases and tablet stands, a clinician Windows tablet (e.g., Microsoft Surface Pro 7 with an Intel core i5 processor), a Polar H10 heart rate monitor, a wireless router, and a speaker. The system uses a single-server multi-client network model where the clinician interface acts as a server, and communicates with the six interactable tablet-buttons, or clients, *via* TCP/IP, using a custom communication protocol. The wireless router allows *R2Play* to use a local Wi-Fi network, thus ensuring it is not dependent on infrastructure Wi-Fi. During the assessment, the clinician interface sends relevant commands to the tablet-buttons (including the character to display and the color of the button), plays appropriate sounds (scramble tone, auditory interference noise, exercise cue, etc.), and receives information from the tablets each time the athlete touches their screens. Heart rate data are streamed into the clinician interface by means of a custom WPF desktop application which communicates with the Polar H10 heart rate monitor *via* Bluetooth Low Energy, using the standard GATT heart rate profile.

### 3.4 Scoring

Data from tablet-button presses and the heart rate monitor are logged by the clinician interface and displayed live as the athlete completes each level. The system quantifies:

• Number of errors: Instances where the wrong button in the sequence is pressed.
• Completion time: How long it takes to complete a repetition of a given level. This is calculated relative to the number of buttons the athlete must press (e.g. number-letter trail would have six button presses, while the Go/No-Go level would have four button presses), and all trails are standardized to be approximately the same distance of path travelled by the athlete.
• Heart rate: ECG heart rate streamed from a Polar H10 heart rate monitor worn by the athlete.

**Figure 4:**
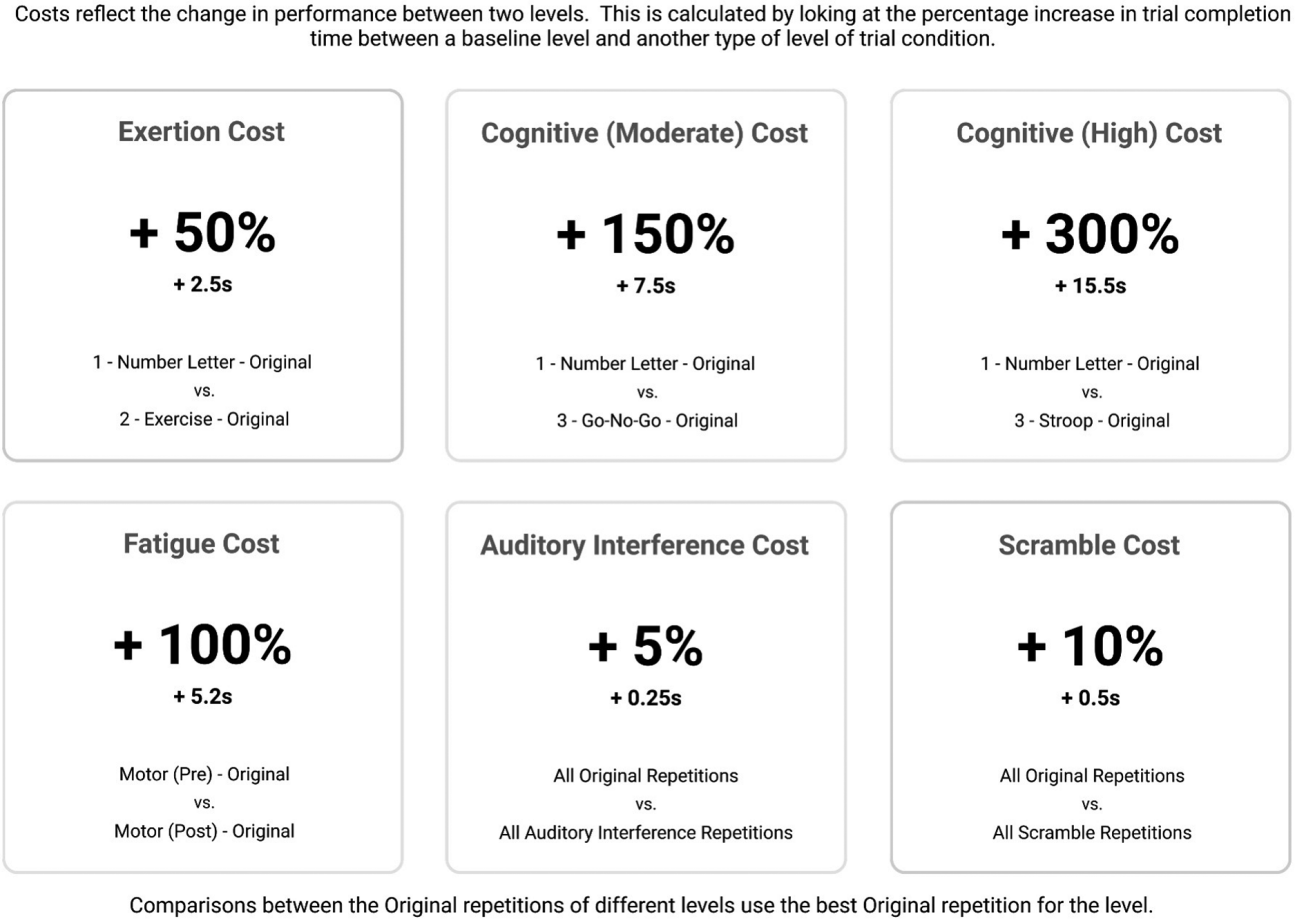
Example cost summary for *R2Play*.

Using these data, the system calculates a series of “costs” that reflect the change in performance between two levels or conditions. This analysis is aligned with traditional dual-task cost equations, where cost is calculated by computing the percent increase in completion time between a baseline level and another more challenging level. In this way, the *R2Play* system can be used to explore the “cost” of physical exertion (exertion trail vs. number-letter trail), cognitive processing (Go/No-Go or Stroop vs. number-letter trail), scramble (all scramble repetitions vs. all original repetitions), auditory interference (all auditory interference repetitions vs. all original repetitions), and fatigue (post-assessment motor trail vs. pre-assessment motor trail). Each of these costs is displayed within a “cost summary” in the *R2Play* results (Figure 4). While a larger study is required to understand the sensitivity of the *R2Play* assessment and the interpretation of *R2Play* results, cost analyses have shown promise in simpler dual-task assessments of concussion recovery (20, 52, 53), and in neurological populations outside of concussion (54, 55).

Lastly, the interface also provides input fields to record clinician's notes/observations (e.g., loss of balance) throughout the assessment and athlete's perceived exertion and concussion symptoms after each level. These data are synthesized, and results are presented upon task completion.

## 4. Objective three: Interface testing

Having designed and developed a functional hi-fidelity stakeholder-guided prototype, the next stage of our design-thinking process was to test the system. Of note, evaluating a complex system like *R2Play* is anticipated to span multiple years with ongoing iteration. In this section, we describe the first stage of prototype testing, where we examine the usability of the clinician interface (i.e., the app that clinicians will use to facilitate administration of the *R2Play* assessment) in a streamlined cognitive walkthrough format. A cognitive walkthrough allows a system's designer to pinpoint areas where users are challenged by the interface or encounter usability problems. Through this process, we aimed to address the following questions: (1) Are clinicians able to use and effectively navigate the *R2Play* software interface as measured by the number of recoverable and irrecoverable errors? (2) Are participants satisfied with the *R2Play* interface as indicated on the System Usability Scale, and (3) What modifications or suggestions do clinicians have that will help inform the development of *R2Play*?

### 4.1 Recruitment and sampling

We employed convenience sampling to recruit five clinicians for the cognitive walkthrough. It has been established previously that approximately 75% of usability problems are typically identified by as few as three to five evaluators (56, 57). The inclusion criterion for clinicians was experience assessing children and adolescents with concussion aged 6–19 years.

### 4.2 Study procedure

Cognitive walkthroughs were carried out over the online videoconferencing platform Zoom (44). The researcher (DD) began by opening the *R2Play* interface on their computer, and then shared their screen with each clinician participant and gave them “remote control” of the screen. In this way, the walkthrough procedure was carried out without deploying software to individual participants. To begin, the researcher provided context to the walkthrough by presenting the clinician with an example scenario: the return-to-play assessment of a teenage athlete named Emma. The participant was then required to use the interface to navigate through tasks like “create an athlete profile for Emma” or “begin Emma”s *R2Play* assessment session”. Supplementary Table S3 provides a list of all key tasks included in the *R2Play* cognitive walkthroughs. Participants were also encouraged to “think aloud” to provide real-time feedback as they explored the interface, commenting on features that they liked, disliked, or found confusing.

### 4.3 Measures

Video of the screen recording and audio of the participant “thinking out loud” were recorded. After task completion, the System Usability Scale (SUS) was administered online as a subjective assessment of overall usability (58). This scale consists of ten five-point Likert questions and is a widely used validated measure of perceived usability, which allows researchers to rapidly appraise user experience (59). On this scale, systems with a score of over 68% are deemed to have above average usability (60). Each clinician also participated in a semi-structured exit interview where they were prompted to share any further comments on the interface, suggestions for changes, and discussions about any obstacles or difficulties with task completion.

### 4.4 Data analysis

A standardized observation checklist was developed to help document task performance, usability problems, and their severity. If the participant was unable to complete a task without help, this was classified as a severe usability problem. In some instances, participants recovered from errors on their own during the session and were eventually able to complete their tasks. These errors, documented as recoverable, were still considered as usability issues that should be addressed. Following review of field notes, screen/audio recordings, exit interviews, and task completion scoring, a copy of each interface screen was annotated to reflect areas where participants had difficulties and suggestions for changes (DD). These changes were then discussed within the broader group (DD, EL, EB, SS). This allowed the research team to compile a prioritized list of usability problems to address. The SUS score for the *R2Play* interface was also computed to quantify the usability of the system.

### 4.5 Results

Demographic information for the 5 clinician participants is shown in Table 3.

**Table 3:** Cognitive walkthrough participant characteristics.

Age Range	Gender	Discipline	Sector	Use of technology in practice?
45–54	M	Athletic Therapist and Kinesiologist	Private	Once per week.
45–54	F	Physiotherapist	Private	Every day or almost every day.
25–34	F	Occupational Therapist*	Public	Once per week.
25–34	F	Specialized Neurorehabilitation Athletic Therapist and Physiotherapist*	Private	Every day or almost every day.
45–54	M	Athletic Trainer	Private	Every day or almost every day.

* Two clinicians who took part in the earlier *R2Play* feedback interview expressed interest in providing more feedback on the project and were included in the cognitive walkthrough sample.

The *R2Play* interface scored an average of 81% (SD = 8.02) on the SUS, placing it in the “good” to “excellent” usability range (60). This positive result was reflected in the analysis of cognitive walkthrough data, as feedback was largely related to refining layout and tweaking details, as opposed to serious usability problems. Participants seemed comfortable with navigating the *R2Play* interface and found the “flow” easy to follow. Specific feedback was given regarding the layout of the home screen and assessment screen, as well as the wording of the post-assessment check-in screen. Multiple participants made recoverable errors, such as clicking on areas of the screen that were not enabled as buttons, or had difficulty locating buttons including “create profile” and “start assessment”. No unrecoverable errors (i.e., terminating the assessment) were made during the cognitive walkthroughs. Following walkthroughs, the research team created an updated set of wireframes to depict design changes including adjustments to wording, font size, and screen behavior.

## 5. Discussion

The goal of this project was to work collaboratively with stakeholders to develop a prototype of a multi-domain assessment to assist with return-to-play decision making after pediatric concussion. To this end, our research team leveraged a variety of methodological and design strategies, which were guided by an overarching design-thinking framework. This type of user-centered approach is well-suited for the type of complex, multifaceted problem that building a novel return-to-play assessment encompasses (30).

### 5.1 Key lessons learned

This paper documented the development of the *R2Play* system from inception to preliminary prototype testing. We aspired to build an assessment that reflected user needs and could be translated into clinical practice, addressing a gap in current concussion return-to-play protocols. We would like to highlight not only the system itself, but also two key takeaways from our research process: the benefits of a user-centered approach to design, and the potential of technology to bolster clinical assessment.

#### 5.1.1 The *R2Play* system

Multi-domain paradigms implemented in research are commonly dual-task assessments, consisting of a simple gait condition paired with an easy-to-explain cognitive task (e.g., walking while subtracting serially by sevens). The *R2Play* system, on the other hand, taxes youth athletes through multi-sensory stimuli both physically and cognitively in a way that strives to be more sport-like. Athletes must respond to a dynamic environment and carry out a series of increasingly complex cognitive tasks, all while exerting themselves physically. This aligns with best practice guidelines for return-to-play and may provide clinicians with richer clinical data due to the complexity of integrating across domains simultaneously, as opposed to in silos (18, 19).

#### 5.1.2 User-Centered approach to design

Our system development was informed by feedback from clinicians and youth sports coaches through multiple iterations of design. In doing so, we aimed to identify and circumvent barriers to *R2Play* adoption from the outset and build a prototype that reflects the needs of end-users. Key criteria that were considered in the *R2Play* prototype design were that it should be: sport-like, fun for youth athletes, easy-to-use, low-cost, flexible, and clinically informative.

As a result of our user-centered design process, the *R2Play* system has a drastically different look and feel than current dual-task return-to-play assessments used predominantly in research (18, 19, 24). While common dual-task assessments prioritize experimental control by creating highly constrained testing environments, the *R2Play* task is designed to feel sport-like in terms of difficulty and skills (24).

Although seeking user feedback added to the initial workload of system development, it allowed us to move forward with a flexible concept that was firmly grounded in the feedback of end-users. Because of this early investment, refinements made later down the line were mostly minor changes. We also have a wealth of information extracted from interviews to guide next steps for iterative refinement of the *R2Play* system in a way that reflects the priorities of stakeholders. Our valuable experiences in *R2Play* development reflect the broader user-centered design literature, which point to seeking stakeholder engagement throughout the research process as a highly beneficial undertaking (30, 61).

#### 5.1.3 Applications of technology to bolster clinical assessment

The development of *R2Play* also sets an example for future work in health and rehabilitation. We have highlighted one way for low-cost technology to potentially support and fill a gap in clinical assessments when thoughtfully designed. The *R2Play* technology was chosen with a focus on *flexibility* and *low-cost* to eliminate possible barriers to clinical implementation. In our system, the tablet-buttons have a dual-purpose: they display the trail stimuli *and* assist in scoring the task. This sets the *R2Play* system apart from other multi-domain assessments, which are typically scored through motion capture or inertial sensors (24). While these scoring methods are useful for research, they are not readily available or amenable for use in clinical settings.

By logging button presses within the *R2Play* system, we are able to capture metrics like time to completion and errors, which can then be compared through “cost” analyses (see “scoring” section). Further work is being done to incorporate low-cost wearable sensors into the *R2Play* scoring system.

### 5.2 Limitations and next steps

The system described here is an early prototype. Limitations to our design process exist, as we had relatively small samples for stakeholder feedback interviews and did not include youth athlete feedback in the initial design process of *R2Play*. It is important to note, however, that youth athletes will be continuously engaged with for feedback during the ongoing testing and refinement of the *R2Play* system prototype (currently in progress). Due to challenges in recruitment (likely aggravated by the COVID-19 pandemic), our sample of youth sports coaches was smaller than targeted. As a result, the study may be lacking perspectives from lacrosse, field hockey, or volleyball coaches, for example. This work merits expansion through future studies, as bolstering the ecological validity of multi-domain assessment through simulating sport was a central goal of our work. One possible avenue to capture a broader concept of “sport” and critical sports skills would be allowing a breadth of youth sport coaches to observe piloting of the *R2Play* assessment with healthy youth athletes to garner feedback on its relevance to their sports.

Next steps for *R2Play* include testing the system in its entirety with clinicians and healthy youth athletes to develop an understanding of whether our design objectives (Table 1) were met, if it is feasible in practice, and areas in need of refinement. Through this testing, we anticipate further iterations to *R2Play* based on the perspectives gained from youth athletes regarding whether *R2Play* is fun, appropriately challenging, and sport-like. Work must also be done to support clinicians in implementing the *R2Play* system, by developing training resources and validating the scoring of the *R2Play* assessment.

During interviews, clinicians and youth sports coaches also expressed excitement that *R2Play* could be translated to populations outside of concussion, including moderate-severe brain injuries, other types of sports injuries, or as a training tool for healthy athletes. The potential for *R2Play* to positively impact other populations also bears investigation in the future.

## 6. Conclusion

The current paper has described the first steps in the development of the *R2Play* system, summarizing a two-year research program which sought to build a novel multi-domain return-to-play assessment for youth athletes following concussion. This process was guided by the following core objectives: 1) to capture end-user perspectives, define our problem, and ideate solutions; 2) to build an *R2Play* system prototype; and, 3) to test the *R2Play* interface. To address these, we integrated various methodological and design strategies: from qualitative interviews, to iterative prototyping, to cognitive walkthroughs. Ultimately, we have described the design process behind the *R2Play* system and highlighted the strengths of a user-centered approach and potential applications for technology to support clinical practice. Through this work, we hope to establish a new standard of care for youth athletes after concussion, by implementing an assessment that is designed with their activities and needs in mind. In the future, *R2Play* will help promote the safety of youth athletes and allow clinicians, youth, and their caregivers to have confidence when returning to play following a concussion.

## Data Availability

Data will be made available by the authors on request and in line with ethical standards of practice and institutional data transfer agreements.
