# Supplementary material for: R2Play development: Fostering user-driven technology that supports return-to-play decision-making following pediatric concussion
Source: Front Rehabil Sci. 2022 Dec 5;3:1051579. doi: 10.3389/fresc.2022.1051579 (PMC9760755; doi:10.3389/fresc.2022.1051579)
Supplement: Supplementary file 1 [file Table1.docx]

Supplementary Material

# Supplementary Tables

Supplementary Table 1. Feedback Interview Design Table.

| Theme | | Category | Sub-Category | Description | Feedback  (*Implemented, iterating, or incompatible)* |
| --- | --- | --- | --- | --- | --- |
| 1. Individuals |  | Accessibility | Wheelchair Accessibility | Adapting *R2Play* for wheelchair users. | *Implemented:* Moved tablets from being on the ground into elevated stands, so that wheelchair users would not have to bend down to press tablets. |
|  |  |  | Color Blindness | Adapting *R2Play* for color blindness. | *Iterating:* For Go/No-Go levels could put an outline of a stop sign around the stimulus in future iterations. |
|  |  |  | Dyslexia | Adapting *R2Play* for dyslexia. | *Iterating:* Possible to use the color trails test or the shape trails test to mitigate the impact of dyslexia. |
|  |  |  | Language | Adapting *R2Play* for non-English speakers. | *Iterating:* Translation for different languages to be included in future iterations of *R2Play*. |
|  |  | Individual Context |  | Gathering a thorough history so recovery can be compared against their individual “normal”. Factors to consider include psychosocial influences, school, symptoms before/ after, mental health, sport training profile (experience & level of play), co-morbidities. | *Implemented:* Created an optional clinical history which touches on these factors. To be used at beginning of first session if desired. |
|  |  | Age |  | Adapting *R2Play* for younger children. | *Iterating:* Possible to use the color [56] or the symbol trail making test [57] for younger children. |
| 2. *R2Play* System | 2.a. Task | Perception-Action Integration |  | Navigating self in relation to moving/ changing stimuli. Dynamic decision-making reactions to perceived stimuli. | *Implemented:* Brainstormed and implemented a “scramble” condition where nodes change places midway through the trail, cued by an auditory stimulus. |
|  |  | Spatial Awareness |  | Add an obstacle that they have to navigate around to tax spatial awareness. | *Iterating:* At this time, we are unsure how to incorporate this feedback in a safe way. Will be considered for future iterations of *R2Play*. |
|  |  | External Perturbation |  | Test coordination and confidence by having participants take contact (ex: through a bump or a jostle by clinician). | *Iterating:* At this time, we are unsure how to incorporate this feedback in a safe way. Will be considered for future iterations of *R2Play*. |
|  |  | Auditory interference |  | Sports take place in a noisy, stimulating environment. | *Implemented:* An auditory interference condition has been incorporated that simulates the background noise one might hear while playing sports. Will help us better simulate a sports environment. |
|  |  | Customization | Sport-Specific Skills | Adding in elements of specific sports (dribbling a basketball, dribbling a soccer ball, carrying a hockey stick, catching/ passing a ball). | *Iterating:* Possible to implement in future iteration of *R2Play* where the assessment is customizable for specific sports. Could add implements (balls, sticks, etc.) and customize the level in terms of duration (mirror the length of a shift) and size (larger spaces/ longer distances for sports with larger playing fields). |
|  |  |  | Target Size | Could change the size of the target displayed on the tablet-buttons to make the task easier or harder. | *Iterating:* The system can support this, and it may be integrated in future iterations. |
|  |  | Working Memory |  | Could have athletes memorize a sequence of numbers and complete the trail. | *Iterating:* It is possible to implement a digit span task with the current system, however this was not included at this time because we aimed to keep the assessment short. Working memory tasks also pose a problem for creating longer (more physically fatiguing) sequences, as you cannot go above 7 +/- 2 items. |
|  | 2.b. Interactive Buttons | Durability |  | People may step on/ trip over tablets. | *Implemented:* Integrated tablet cases and stands. |
|  |  | Virtual Reality (VR) |  | Possible application for VR. | *Incompatible:* Not compatible with current system. We chose not to use VR technology due to confounding symptoms of VR sickness/ concussion. Suitable for more static use as opposed to a dynamic exertional assessment like *R2Play*. |
|  | 2.c. Interface | Recording Observations |  | Ability to note observations as the participant runs through the assessment. | *Implemented:* added a “notepad” on the assessment screen in the interface. |
|  |  | Kid-Friendly Performance Summaries |  | Ability to display results and use them as a tool to communicate with athlete to show progress and explain rationale. | *Implemented:* A graphic summary of results was developed with young athletes in mind. |
|  | 2.d. Scoring | Normative data |  | Normative data for interpretation of results: age/ height normed data. | *Iterating:* Information is collected in “client profile” includes age, height, comorbidities, etc. Goal being to create a normative data set in the future. |
|  |  | Other Errors | Movement Patterns | Contextualizing slow completion times. Ex: Did they run at a button and not press it? Were they freezing or hesitating? | *Iterating:* Incorporated as “movement patterns” in the experimental *R2Play* scoring system using wearable accelerometers. Aim is to give context to completion time. Sensor validation study is underway, and it is possible that these metrics will be included in future iterations of *R2Play.* |
|  |  |  | Physical Errors | Recording trips, falls. | *Implemented:* Able to make a note of these behaviors in the notepad on the assessment screen. |
|  |  |  | Reaction Time | Quantifying reaction time as a measure of performance on the R2Play task. | *Iterating:* Algorithms for detection of reaction time using wearable accelerometers are currently being developed and refined. Possible to incorporate these metrics in future iterations of *R2Play*. |
|  |  |  | Path Length | Use of a scoring system that compares speed of movement and path length. | *Iterating:* Currently this could be implemented using a local positioning system that measures location through an ultra-wideband tracking hub. However, this technology is too expensive and would create a barrier to accessibility of the R2Play system. The experimental scoring with accelerometers aims to fill this gap. |
|  |  | Heart rate Zones |  | Provide targeted heart rate zones for exertion during levels. | *Iterating:* A larger study is needed to understand the levelling of *R2Play* and how athletes respond to the task before prescribing target heart rate zones. |
| 3. Clinic integration |  | Assessment Space |  | How large does the assessment space need to be for the R2Play task to be appropriately challenging? How to balance out the difference between doing it in a large vs. small space? | *Iterating:* System has built-in flexibility to work in different spaces. Optimal layout to be determined through the feasibility study and future investigations. |
|  |  | Assessment Time |  | Clinicians would like to spend less than 30 minutes on this assessment. | *Implemented:* Streamlined *R2Play* level structure to shortened assessment time. |
|  |  | Training |  | The importance of training clinicians to use and interpret R2Play properly. | *Iterating:* Currently, there is no training manual for *R2Play*. As the system is refined, training resources will be developed for interpretation and implementation of the assessment. |

Supplementary Table 2. Semi-Structured Interview Guides

| **Clinician Interview Guide** | |
| --- | --- |
| **Focus area** | **Example comments and questions** |
| Introductory Questions | -What experience do you have with assessing RtoP in your practice? How long ago was this experience?   - In your practice can you describe to me the “flow” of the clients through the RtoP process? Who was conducting the RtoP assessment (physio, OT, NP, physician)? - What assessment tools do you typically use?   -Did you feel confident in the RtoP assessments that you use?   - Would you describe this experience as positive or negative? Both? What would you say shaped that opinion?   -Do you use any technology in practice to assist with the assessments? Any apps or technology in general in your practice? |
| *Introduction to R2Play* | |
| Initial R2Play feedback | -What do you think about our idea for the R2Play multitask assessment? Do you have any thoughts on it?   - Does this look like something youth athletes could do? - Do you think this could be an informative assessment? A fun assessment for youth athletes? - Does this emulate the skills you think are important in sports? |
| Design considerations | - In your experience, are there things you think are important to consider when we’re designing technology to support RtoP assessments?   - Which of these do you think are most important? |
| Barriers for adoption | - What challenges would you foresee in using this kind of technology in the real world?  - Which of these do you think will be the biggest challenges?  -Could you implement the R2Play assessment in your practice? Why or why not?  -What do you think you/ your practice could pay for a system like ours? |
| Skills in multi-task assessment | - Playing sports tests an athlete’s endurance, decision-making, sensory processing, and so much more  - What skills do you think are most important to assess in a multi-task environment for RtoP decision-making?  -Are there any skills that you see youth athletes often have difficulty with when returning to sports? |
| Feedback questions | - How long should the assessment be?  - What information would you want the technology to collect/provide during and after the assessment?  -Who do you see carrying this out?  - Are there any other tools that you would like to have access to during the assessment (that you may not have access to now)?  -How much space would you have to conduct an assessment like R2Play? |

| **Coach Interview Guide** | |
| --- | --- |
| **Focus area** | **Example comments and questions** |
| Introductory Questions | - What has been your experience with concussion and RtoP in your sport? How long ago was this experience?  - Can you give me an understanding of your coaching experience/ resume?  -Did you feel confident in the RtoP assessment when your player returned?   - Would you describe this experience as positive or negative? Both? What would you say shaped that opinion? |
| *Introduction to R2Play* | |
| Initial R2Play feedback | -What do you think about our idea for the R2Play multitask assessment? Do you have any thoughts on it?   - Does this look like something youth athletes could do? - Do you think this could be a fun assessment for youth athletes? - Does this emulate the skills you think are important in sports? Does it seem sport-like to you? |
| Skills in multi-task assessment | - What skills do you think are most important to assess in a multi-task environment for RtoP decision-making?  -Are there any skills that you see youth athletes often have difficulty with when returning to sports?  -If you were to design/ choose a drill to see if someone was ready to come back to play, what drill would you choose? |
| General Sports Skills | -What skills are important in your sport?  -Are there skills you think are important across all sports?  -What kind of drills do you use to train these skills?   - Any drills that are especially cognitively taxing?   -Does this assessment remind you of your sport/ simulate skills from your sport?  -Does this assessment remind you of sports in general? |
| Barriers for adoption | - What challenges would you foresee in using this kind of technology in the real world?  - Which of these do you think will be the biggest challenges? |

Supplementary Table 3. Key Tasks for R2Play Cognitive Walkthroughs.

| Task |
| --- |
| - - 1. Access athlete library and open an athlete profile |
| - - 1. Start an *R2Play* session |
| - - 1. Create an athlete profile for a fictional client |
| - - 1. Create a new fictional assessment room |
| - - 1. Connect tablets |
| - - 1. Personalize *R2Play* assessment session for an intermediate athlete |
| - - 1. Complete a post-concussion symptom inventory for a fictional client |
| - - 1. Begin an *R2Play* assessment session (watch video example of level) |
| - - 1. Report how long it took the client to complete the audio interference condition of the letter-number trail |
| - - 1. Report how the participant’s cognitive costs are calculated |
| - - 1. Explore the supplementary results graphs |
| - - 1. Add a clinical impression and recommendation |
| - - 1. Export results |
| - - 1. Navigating interface- return to home |
